# The Role of Astrocytes in Poststroke Rehabilitation

**DOI:** 10.1002/brb3.70551

**Published:** 2025-06-04

**Authors:** Ying Han, Li Huang, Jiaojiao Wu, Guiling Wan, Linhong Mo, Aixian Liu

**Affiliations:** ^1^ Neurological Rehabilitation Center, Beijing Rehabilitation Hospital Capital Medical University Beijing China

**Keywords:** astrocyte, ischemic stroke, neuron, rehabilitation

## Abstract

**Introduction:**

Ischemic stroke is a leading cause of death and chronic disability worldwide. Therapeutic options to reduce stroke‐related disability are still limited. Resident astrocytes are the most abundant glial cells in the brain and respond to the pathophysiological changes induced by ischemic stroke.

**Methods:**

Comprehensive literature search and narrative review to summarize the current field of astrocytes in poststroke rehabilitation using the PubMed database.

**Results:**

This review first presents that astrocyte activation is critical for scar formation, synaptogenesis, synaptic remodeling, inflammation limitation, and metabolic regulation. Next, we summarize the contributions of astrocytes to neural repair after stroke with a focus on their effects on exercise, transcranial magnetic stimulation (TMS), transcranial electrical stimulation (tES), music therapy, acupuncture, hyperbaric oxygen therapy (HBOT), and enriched environment (EE).

**Conclusions:**

In this review, we comprehensively analyze the role and possible mechanisms of astrocytes in various rehabilitative treatments and suggest that targeting astrocytes may be an effective strategy for the development of innovative therapeutic strategies for ischemic stroke.

## Graphical abstract

Contributions of astrocytes to neuroprotection in different types of rehabilitation after ischemic stroke.

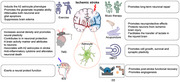

## 1 Introduction

Stroke is the second leading cause of death and the leading cause of disability in adults worldwide. Survivors suffer long‐term disability and require extensive rehabilitation (Barthels and Das 2020). Ischemic stroke, the most common type of cerebrovascular disorder, is caused by an abrupt blockage of an artery that restricts blood flow (Campbell et al. 2019). Ischemic stroke can cause a range of pathophysiological changes in nerve cells, including neuronal death (Tuo et al. 2022). Restoring central nervous system (CNS) function after stroke has long been a major goal of neuroscience research. Astrocytes, the most abundant cells in the CNS, represent approximately 20%–40% of brain cells. On the basis of morphology and specific protein markers, there are two major types of astrocytes in the adult brain: fibrous astrocytes, which are found in white matter tracts, and protoplasmic astrocytes, which are found in gray matter. Glial fibrillary acidic protein (GFAP) is the hallmark intermediate filament protein of astrocytes and is considered a “pan‐astrocyte” marker (Baba et al. 1997; Hol and Pekny 2015). Aldehyde dehydrogenase 1 family, member L1 (Aldh1L1) protein is highly expressed in the cell body and extensive processes of an astrocyte, making it a new “pan‐astrocyte” marker (Y. Yang et al. 2011). Astrocytes play a critical role in maintaining control and homeostasis in both health and disease. Astrocytes are supportive glial cell components in neural tissues and play critical roles in neural conduction, regulation of blood flow, synaptic transmission, modulation of metabolism, and formation of the blood–brain barrier (BBB) (Bonvento and Bolaños 2021; Song et al. 2021{Song, 2021 #46}; Stogsdill et al. 2017). Thus, astrocytes are promising targets for neural repair and remodeling after ischemic stroke.

## 2 Methods

References for this review were identified through a systematic search of PubMed up to May 2024, supplemented by backward citation tracking. The search strategy combined terms related to stroke rehabilitation (e.g., “stroke”) and therapeutic interventions targeting astrocyte‐mediated mechanisms, including exercise, repetitive transcranial magnetic stimulation (rTMS), transcranial electrical stimulation (tES), music therapy, acupuncture, hyperbaric oxygen therapy (HBOT), and enriched environment (EE). Specific search terms were structured as follows: (1) “stroke” AND “astrocyte” AND “exercise”; (2) “stroke” AND “astrocyte” AND “repetitive transcranial magnetic stimulation (rTMS)”; (3) “stroke” AND “astrocyte” AND “transcranial electrical stimulation (tES)”; (4) “stroke” AND “music”; (5) “stroke” AND “astrocyte” AND “acupuncture”; (6) “stroke” AND “astrocyte” AND “hyperbaric oxygen therapy”; and (7) “stroke” AND “astrocyte” AND “enriched environment.” This narrative review included articles published between 1983 and May 1, 2024, with a total of 111 references. Of these, 51 articles (45.9%) were published in the last 5 years (2020–2024), including the most recent publication on May 1, 2024. The retrieved literature included different formats: original articles, reviews, randomized controlled trials (RCTs), meta‐analyses, and clinical trials. Studies involving both clinical populations (e.g., patients with diagnosed stroke) and nonclinical controls were included, as well as preclinical research using animal models (e.g., mice and rats). All selected articles were peer‐reviewed and published in English.

## 3 Astrocyte Activation and Its Role in Ischemic Stroke

Under pathological conditions, astrocytes undergo a variety of phenotypic and functional changes, known as reactive astrogliosis. Reactive astrocytes can be identified by increased expression of GFAP and many other proteins as well as dramatic morphological changes. Morphologically, reactive astrocytes become hypertrophic with large processes. After prolonged ischemic stroke, the morphology of reactive astrocytes remains stable, resulting in the formation of a glial scar. Reactive astrogliosis and glial scar formation eventually lead to significant tissue remodeling and permanent structural changes in the penumbra. Recent data suggest that astrocyte activation after ischemic stroke has both beneficial and detrimental effects. For many years, severe reactive astrogliosis and glial scarring at sites of CNS trauma were thought to be barriers to axon regeneration and not conducive to recovery from nerve injury (Silver and Miller 2004). However, recent studies have shown that glial scar formation may gradually reduce the spread of inflammation and lesion volume to prevent brain injury after stroke (Clain et al. 2024; R. Zhang et al. 2018). Reactive astrocytes are heterogeneous, and astrocyte polarization is a new concept in which astrocytes are classified as A1 (neurotoxic) or A2 (neuroprotective) (Escartin et al. 2021; Y. X. Liu et al. 2022). A1 astrocytes can be induced by microglia, and complement component 3 (C3) is a specific marker of A1 astrocytes (Fei et al. 2022). A1 astrocytes lose their normal functional properties and secrete unknown neurotoxins, and activation of the NF‐κB pathway in A1 astrocytes contributes to inflammatory responses and exerts deleterious effects (X. Xu et al. 2018). A2 astrocytes are predominantly induced in ischemic stroke patients and animal models and play a crucial role in synaptic plasticity and brain repair by secreting neuroprotective substances. The JAK/signal transducer and activator of transcription 3 (STAT3) pathway mediates A2 astrocyte activation, including glial scar formation, reduction of leukocyte infiltration, and secretion of cytokines (Z. B. Ding et al. 2021; Justicia et al. 2000).

Ischemic stroke causes changes in the morphology, metabolism, and gene expression of reactive astrocytes. Reactive astrocytes can be identified by morphological changes, becoming hypertrophic with enlarged processes that can be visualized by GFAP staining. Following stroke, astrocytes can provide essential metabolic support to neurons during focal cerebral ischemia, and ATP can be released from activated astrocytes (Cho et al. 2022). In addition, the astrocyte–neuron lactate shuttle and the expression of functional transporters for glutamate and GABA uptake are altered in reactive astrocytes. Reactive astrocytes can also secrete mediators relevant to the protective functions of astrocytes, including neurotrophic, growth, and anti‐inflammatory factors (Caglayan et al. 2023). In addition, astrocyte‐derived metalloproteinases (MMPs) (Y. Zhang et al. 2013), metabolites (Bonvento and Bolaños 2021), and extracellular matrix (ECM) (Johnson et al. 2015) proteins can promote neuronal plasticity following CNS insult. Thus, understanding the regenerative potential of astrocytes is fundamental for the development of strategies to restore neurological function after ischemic stroke.

## 4 Roles of Astrocytes in Stroke Rehabilitation

### 4.1 Astrocytes in Exercise

Exercise has been shown to be neuroprotective in both clinical and animal studies, reducing infarct volume, improving cerebral edema, mitigating BBB dysfunction, and increasing survival rates (Ahn et al. 2017; Di Raimondo et al. 2020; Y. H. Ding et al. 2006). Exercise can increase the expression of GFAP, a prototypical marker for the immunohistochemical identification of astrocytes, as well as the density of astrocytes in the brain (Dutra et al. 2012; Saur et al. 2014). Furthermore, morphological changes in astrocytes are associated with increased brain‐derived neurotrophic factor (BDNF) and postsynaptic density protein‐95 (PSD‐95) in astrocytes after exercise (Belaya et al. 2020). In addition, exercise induces changes in astrocyte polarization in healthy animals. Exercise alters astrocytic morphology by increasing the degree and length of ramification and inducing polarization of astrocyte endfeet, thereby affecting the area of contact with capillaries (Saur et al. 2014). Astrocyte endfeet envelop the cerebral capillaries that form the BBB, and astrocytes can take up glucose from the blood (Magistretti 2006).

Glial metabolic plasticity is associated with synaptic plasticity. Exercise can promote plasma glucose uptake via 17β‐estradiol (E2), kinases, and glucose transporter type 4 (GLUT4) expression (Oosthuyse et al. 2023). E2 can be produced by neurons and astrocytes in the brain, and astrocyte‐derived E2 is induced after brain injury/ischemia and plays a key role in reactive gliosis (Brann et al. 2022). Furthermore, astrocyte‐derived E2 has been shown to be critical for induction of the A2 astrocyte phenotype and activation of the JAK‐STAT3 pathway in a mouse model of ischemic stroke (J. Wang et al. 2020). The evidence from these studies suggests that astrocyte‐derived E2 plays a critical role in the metabolism and neuroprotection of exercise training in patients with ischemic stroke.

Exercise preconditioning protects the brain against ischemic injury by regulating a number of molecules within astrocytes, such as glutamate transporter 1 (GLT‐1), heat shock protein 20 (HSP20), and aquaporin‐4 (AQP4). The expression of GLT‐1, a glutamate transporter, is significantly reduced after ischemic stroke (Yeh et al. 2005). Research has shown that pre‐ischemic exercise increases GLT‐1 levels (X. Yang et al. 2012) and regulates glutamate receptors (F. Zhang et al. 2010). Increased astrocytic GLT‐1 is associated with promoting the reuptake of glutamate, thus exerting a neuroprotective effect in ischemic stroke. In addition, HSP20 belongs to the small HSP family and has multiple functions. Upregulation of HSP20 expression in the heart has been shown to protect against cardiac ischemia/reperfusion (I/R) injury (Qian et al. 2009). Research has shown that exercise preconditioning improves outcome in ischemic stroke and attenuates both neuronal and glial apoptosis by promoting HSP20 expression in neurons and glial cells (Lin et al. 2015). AQP4 is the most abundant water channel protein in the brain. Increased expression of AQP4 in the brain results from infarction, which may be related to the development of cerebral edema (Murata et al. 2020). Exercise reduces astrocytic AQP4 expression in the ischemic core by attenuating the decrease in IL‐1 receptor antagonist (IL‐1RA) expression, an anti‐inflammatory cytokine that suppresses brain edema, following ischemic stroke (Gono et al. 2023).

Exercise increases the interaction between astrocytes and other neural cells (such as neurons, microglia, and endothelial cells [ECs]) (Li et al. 2021). Astrocytes release several neurotrophic factors (NTFs) and gliotransmitters that act at nerve synapses, including IGF‐1, BDNF, glutamate, acetylcholine, ATP, and lactate, which are involved in the remodeling of astrocyte–neuron crosstalk (Durkee and Araque 2019; Stogsdill et al. 2017). Exercise mediates crosstalk between astrocytes and microglia via their secreted molecules, such as glial cell line‐derived neurotrophic factor (GDNF), cerebral dopamine neurotrophic factor (CDNF), and BDNF, which are secreted by astrocytes and are involved in modulating microglial activation (Rocha et al. 2012). In addition, a number of studies have shown that astrocytes maintain the integrity of the neurovascular unit (NVU) and mediate BBB permeability by influencing ECs (Benz and Liebner 2022). Thus, astrocyte–neuron–microglia–EC interactions may be induced by exercise and contribute to neuroplasticity.

### 4.2 Astrocytes in Physical Therapies

#### 4.2.1 Astrocytes in Transcranial Magnetic Stimulation (TMS)

TMS is widely used in clinical rehabilitation and has a direct effect on neuronal excitability; glial cells can also respond directly or indirectly to electrical activity. Astrocytes are important regulatory cells and are likely to be critical mediators of TMS‐induced neural plasticity. TMS affects astrocytes, and 10 Hz magnetic stimulation of cultured astrocytes induces a transient increase in the expression of GFAP (Chan et al. 1999). Another study showed that 1 Hz repetitive magnetic stimulation (rMS) increases intracellular calcium in primary astrocytes and that calcium can respond to different stimuli. In addition, rMS can affect astrocyte physiology by promoting migration and proliferation of primary astrocytes in vitro (Clarke et al. 2017).

Astrocytes are likely to influence synapse formation and spine shape in response to TMS. Neurotransmitter release from neurons is sensed by astrocytes and the subsequent release of gliotransmitters; thus, astrocytes may be the cellular effectors of TMS. rTMS can modulate astrocytes, increase axonal density, and promote neural plasticity after oxygen and glucose deprivation followed by reperfusion (OGD/R) injury, and the expression of synaptic markers, such as PSD‐95, calcium/calmodulin‐dependent protein kinase II (CaMKII), synapsin I, and synaptophysin, could be upregulated (Hong et al. 2020). In addition, high‐frequency rMS (HF‐rMS) directly stimulates the release of several trophic factors by astrocytes, and GDNF is one of the released factors that contributes to neuronal protection. The presence of astrocytes helps maintain the number and length of neurites and increases the number of neurons, and GDNF plays a critical role in the beneficial effects induced by HF‐rMS (Gava‐Junior et al. 2023; Roque et al. 2021). In cortical neuron‐glia cultures exposed to OGD/R, the application of HF‐rMS increases the number of cells expressing extracellular signal‐regulated kinase‐1/2 (ERK‐1/2) and c‐Fos, which are thought to be associated with neuronal proliferation and differentiation and the inhibition of apoptosis (Cruz‐Mendoza et al. 2022; Sahu et al. 2021).

Moreover, rTMS improves functional recovery by modulating astrocytic polarization in ischemic stroke (Zong, Li, et al. 2020). Continuous rTMS has been shown to effectively induce an A1 to A2 shift in vascular‐associated astrocytes and increase the levels of platelet‐derived growth factor receptor beta (PDGFRβ) levels associated with A2 astrocytes in a rat photothrombotic (PT) stroke model (Zong, Li, et al. 2020). The potential beneficial effects of rTMS are associated with robust suppression of reactive micro/astrogliosis, proinflammatory cytokines, oxidative stress, and oxidative neuronal damage. Recent work has shown that rTMS treatment suppresses the production of proinflammatory cytokines such as interleukin‐1β (IL‐1β), interleukin‐3 (IL‐3), interleukin‐6 (IL‐6), interleukin‐17 (IL‐17), and tumor necrosis factor alpha (TNF‐α) in an experimental PT rat model of ischemic stroke (Zong, Dong et al. 2020). rTMS inhibits astrocytic secretion of TNF‐α but promotes the ability of interleukin‐10 (IL‐10) to alleviate neuronal death in the context of OGD/R injury (Hong et al. 2020). These findings provide strong evidence for the promising therapeutic effect of rTMS on astrocytes against ischemic neuronal injury following stroke.

#### 4.2.2 Astrocytes in Transcranial Electrical Stimulation

tES, including transcranial direct and alternating current stimulation (tDCS, tACS), is a noninvasive brain modulation technique. tES has been proven to exert beneficial effects on various neuropsychiatric and neurological conditions. tDCS has a neuroprotective effect on the brain after ischemic stroke (L. C. Wang et al. 2021; K. Y. Zhang et al. 2020), and glial cells play a more important role than neurons do in tDCS‐induced brain activity (Ruohonen and Karhu 2012; Saidi and Firoozabadi 2021).

tES can directly modulate gene expression in astrocytes. Research has shown that tDCS increases selenoprotein P (SEPP1) levels in astrocytes and provides neuroprotection by subsequently activating the SEPP1/vesicle‐associated membrane protein 2 (VAMP2)/syntaxin‐4 (STX4) signaling pathway in neurons following cerebral I/R injury in rats (H. Wang et al. 2024). tDCS treatment increases BDNF levels in different regions of the CNS (Filho et al. 2016), and the synthesis of BDNF by astrocytes is well established (Jurič et al. 2008).

Increased levels of astrocytic Ca^2+^ induced by tDCS have been observed in association with cortical plasticity (Monai and Hirase 2016). tDCS‐induced Ca^2+^ increases, which are dependent on the alpha‐1 adrenergic receptor and are diminished by the ablation of noradrenergic neurons, have been identified as being of astrocytic origin. Under tDCS, astrocyte calcium signaling occurs (Monai et al. 2016). tDCS modulates the clearance of metabolic waste in the brain through astrocytic IP3/Ca^2+^ signaling, leading to the modification of delta waves in the mouse brain (Y. Wang and Monai 2024). Additionally, potassium ion (K^+^) flow is independent of neuronal activity and occurs predominantly through astrocytes, which have a high K^+^ conductance at rest (Seifert et al. 2018). K^+^ redistribution may occur via DCS (Gardner‐Medwin and Nicholson 1983), which affects the excitability of neurons and their axon terminals and dendrites. Thus, astrocytes may play an essential role in tDCS‐induced synaptic plasticity via K^+^ and Ca^2+^ in ischemic stroke.

#### 4.2.3 Astrocytes in Music Therapy

Music therapy is a valuable, innovative intervention for stroke recovery, effectively improving motor function (e.g., gait and coordination (Collimore et al. 2023; Y. Wang et al. 2021)), speech rehabilitation (e.g., aphasia recovery (Grau‐Sánchez et al. 2018; Sihvonen et al. 2020, 2021; X. Zhang et al. 2023)), and cognitive‐emotional well‐being (e.g., reducing depression and enhancing motivation (Kiper et al. 2022; Raglio et al. 2017; Sun et al. 2024)) through its unique integration of auditory, rhythmic, and emotional stimulation. Mechanistically, music enhances recovery by promoting neuroplasticity through structural and functional reorganization of motor and auditory networks (Grau‐Sánchez et al. 2013; Raglio et al. 2015; Rodriguez‐Fornells et al. 2012), facilitating sensorimotor integration via rhythmic auditory stimulation to synchronize movement (Sihvonen et al. 2022), and boosting emotional engagement by activating dopaminergic reward pathways to sustain rehabilitation adherence (François et al. 2015). Despite clinical efficacy, cellular mechanisms remain unclear. Recently, a study reveals that music upregulates NTFs (e.g., BDNF) and activates astrocytes (via GFAP expression) in stroke models, enhancing neuronal survival, synaptic repair, and tissue remodeling at lesion sites while modulating inflammation (W. Chen et al. 2021). Future research should refine music‐based therapies to leverage these cellular pathways, advancing personalized, noninvasive strategies to optimize stroke rehabilitation outcomes.

### 4.3 Astrocytes in Acupuncture

Acupuncture has been used in China for thousands of years in two forms: acupuncture (MA) and electroacupuncture (EA). Studies have confirmed its therapeutic effects on poststroke patients (Xiao et al. 2023). Hundreds of acupuncture points are distributed throughout the human body and can be activated by acupuncture needles. Acupuncture‐induced signals are then transmitted to the spinal cord and related brain regions. This mechanism may involve the regulation of neural transmitters and inflammatory factors, thereby exerting neuroprotective effects.

Reactive astrogliosis is a common phenomenon in ischemic stroke and contributes to axonal remodeling and recovery of neurological function after stroke (Z. Liu et al. 2014). EA at the LI11 and ST36 acupoints was shown to exert neuroprotective effects via the activation of GFAP/vimentin/nestin‐positive reactive astrocytes and the expression of BDNF in a rat model of cerebral I/R injury (Tao et al. 2016). Astrocyte activation was significantly reduced after 1 and 2 weeks of EA treatment (J. Y. Wang et al. 2018), and the inactivation of spinal astrocytes contributed to the anti‐allodynic effect of EA (Liang et al. 2016). The expression of astrocytic GFAP and measured factors implicated in inflammation, such as matrix metalloproteinase‐9 (MMP‐9), matrix metalloproteinase‐2 (MMP‐2), IL‐1β, TNFα, and immunoglobulin G (IgG), were reduced in the EA‐ST36 group (Gim et al. 2011). IL‐10, a key cytokine, is mainly synthesized by astrocytes and microglia to suppress excessive inflammatory responses. Another study showed that the anti‐inflammatory cytokine IL‐10 acts as a protective factor in spinal astrocytes during EA‐induced pain (Dai et al. 2019). These results prove that the analgesic effect of EA may be related to a reduction in inflammation in astrocytes.

Many molecules and signaling pathways, such as the endocannabinoid system, are involved in the mechanisms underlying the ischemic tolerance induced by EA. EA pretreatment increases ambient endocannabinoid (eCB) levels and subsequently activates the ischemic penumbral astroglial cannabinoid type 1 receptor (CB1R), leading to a moderate upregulation of extracellular glutamate that protects neurons from cerebral ischemic injury (C. Yang et al. 2021). The crucial role of STAT3 in cell survival and proliferation is widely recognized. STAT3 is involved in the neuroprotection induced by EA pretreatment through CB1Rs after transient focal cerebral ischemia in rats (Zhou et al. 2013).

It is known that brain energy metabolism is also disrupted in cerebral ischemia (T. Zhang et al. 2023), and lactate in astrocytes is considered to be the main energy substrate for surviving neurons during the recovery period after ischemia. Glucose transported into the brain is converted to lactate in astrocytes and then delivered to neuronal cells via a monocarboxylic acid transporter (MCT) (Yamagata 2022). EA treatment induces the activation of lactate metabolism in the resident astrocytes surrounding the ischemic area, leading to the upregulation of astrocytic monocarboxylate transporter 1 (MCT1) expression in rats. This upregulation facilitates the transfer of intracellular lactate to the extracellular domain, allowing its use by injured neurons to ameliorate neurological deficits (Lu et al. 2015).

### 4.4 Astrocytes in HBOT

HBOT has been widely used in poststroke rehabilitation and has a multitude of neuroprotective effects on pathological and physiopathological condition, immunological function, and cellular and molecular mechanisms. Reactive astrocytes play an important role in the acute phase of stroke injury. Repeated HBO treatment leads to a long‐lasting antinociceptive response by inhibiting astrocyte activation (Günther et al. 2005; Zhao et al. 2014). The infarct site is surrounded by activated microglia and astrocytes after the occurrence of stroke, and the activated astrocytes express inducible nitric oxide synthase (iNOS), which is also an “A1” astrocyte‐specific marker (Rao et al. 2024). Massive synthesis of NO can enhance pain generation and further lead to the production of various cytokines and inflammatory factors. Early HBO treatment significantly improves hyperalgesia in rats by inhibiting astrocyte activation and decreasing iNOS and neuronal nitric oxide synthase (nNOS) expression (Y. Ding et al. 2018). Thus, reduced expression of iNOS and nNOS in astrocytes may be an important mechanism by which early HBO helps to alleviate neuropathic injury. HBO treatment inhibits astrocyte expression and the release of inflammatory factors, including TNF‐α and IL‐1β (Y. Ding et al. 2018). The anti‐inflammatory effect of HBOT may be mediated by hyperoxia, which interferes with NF‐κB and IκBα (De Wolde et al. 2021). The molecular mechanisms of HBO have not been fully elucidated. According to some existing studies, HBOT inhibits neuroinflammation via regulation of the NF‐κB/MAPK‐CCL2/CXCL1 signaling pathway in cultured astrocytes (Xia et al. 2022). In addition, HBO preconditioning alleviates inflammation in stroke by transferring resilient mitochondria from astrocytes to primary rat neuronal cells, thereby reducing cell death (Lippert and Borlongan 2019).

Nuclear factor erythroid 2‐related factor 2 (Nrf2) is a key transcription factor for genes regulated by antioxidant response elements. Activation of Nrf2 in astrocytes contributes to ischemic tolerance induced by hyperbaric oxygen preconditioning (J. Xu et al. 2014). Another study showed that compared with the corresponding controls, cultured astrocytes at 21% O_2_ promoted the expression of NTFs, including nerve growth factor (NGF), BDNF, neurotrophin‐3 (NT‐3), and GDNF (Xing et al. 2018), which are essential for cell growth, survival, and synaptic plasticity, compared with corresponding controls.

Abnormal glutamate metabolism in astrocytes induces excitatory neuronal toxicity. Research has shown that HBO exposure increases glutamate levels while significantly decreasing glutamine levels. HBO exposure affects the glutamate metabolism in astrocytes, which is closely related to poststroke epilepsy (Y. L. Chen, Li et al. 2016). HBO exposure regulates adenosine metabolism in astrocytes and inhibits HBO‐induced oxygen toxicity in the CNS, which is important for the generation and transmission of excitability (Y. L. Chen, Zhang et al. 2016). Furthermore, a previous study showed that HBO attenuated the expression of superoxide dismutase 2 (SOD2) in astrocytes, thus contributing to the attenuation of the oxidant/antioxidant imbalance that occurs after brain injury (Parabucki et al. 2012).

### 4.5 Astrocytes in EEs

Clinical epidemiological studies have shown that EEs have direct clinical relevance to neurological and psychiatric disorders. An EE is considered a classic and effective means of rehabilitation in animal stroke models and usually consists of a large cage containing ladders, beams, ramps, and a variety of toys (Kempermann 2019). The mechanism by which an EE promotes recovery of neural function has not yet been clearly elucidated. Compared with a standard environment, an EE provides more physical and social stimuli and facilitates motor, sensory, and cognitive activities. Research has shown that EEs are effective in normalizing dysregulation after stroke. One mechanism by which EEs improve cognitive function may be based on the reciprocal effect of EEs and astrocytes, as astrocytes are responsible for myelin formation through direct interaction with oligodendrocyte lineage cells (Gao et al. 2022). GFAP is a specific marker for astrocytes. EEs increased the number of GFAP‐positive astrocytes in the rat hippocampus after middle cerebral artery occlusion (MCAO) (X. Zhang et al. 2021) and increased astrocyte activity after intracerebral hemorrhage (ICH) (Caliaperumal and Colbourne 2014).

An EE is a promising technique for promoting neural plasticity to aid recovery from stroke (Han et al. 2023). Neural plasticity that occurs during stimulation by an EE plays an important role in the functional stabilization that allows the brain to adapt to environmental changes during development and adulthood. In addition to the high plasticity of neurons, morphological changes in astrocytes have also been observed after exposure to EEs (Markham and Greenough 2004). Further studies support an increased contact between features related to astrocyte morphology and synapse formation in rats (Jones and Greenough 1996). EEs promote synaptic transmission and plasticity (Eckert and Abraham 2013) and communication between astrocytes and synapses by controlling synaptic transmission (Durkee and Araque 2019; Q. Zhang and Haydon 2005). It is well known that astrocytes not only support neuronal activity through their mechanical and structural effects but also promote functional recovery after stroke by secreting cytokines and growth factors. Another study showed that EE increased astrocyte‐derived BDNF and vascular endothelial growth factor (VEGF) expression in rats with ischemic stroke (Guo and Bi 2024). Moreover, EE‐induced astrocyte proliferation and BDNF expression are significantly correlated with the outcome of neural function in rats after MCAO (X. Chen et al. 2017). In conclusion, EEs promote neuroplastic processes and exert a neuroprotective effect via astrocytes in ischemic stroke.

## 5 Conclusions

We have described the multidimensional functions of astrocytes in different rehabilitation modalities after stroke with a focus on enhancing neuroprotection (Table 1). In addition to the rehabilitation methods mentioned in this review, speech and language therapy, cognitive behavioral therapy (CBT), paraffin therapy, massage, traction, herbal fumigation, and psychotherapy are widely used in poststroke rehabilitation. However, the involvement of astrocytes in these therapies has rarely been reported.

**TABLE 1:** Therapeutic targets for astrocyte associated with ischemic stroke.

Treatment types	Mediator	Full name	Species/Model	Roles	References
Exercise	E2	17β‐Estradiol	Global cerebral ischemia (GCI) model in mice	Inducts the A2 astrocyte phenotype	Brann et al. (2022)
	GLT‐1	Glutamate transporter 1	MCAO model in rats	Promotes the glutamate reuptake ability	X. Yang et al. (2012), F. Zhang et al. (2010)
	HSP20	Heat shock protein 20	MCAO model in rats	Attenuates both neuronal and glial apoptosis	Qian et al. (2009), Lin et al. (2015)
	AQP4	Aquaporin‐4	MCAO model in rats	Suppresses brain edema	Murata et al. (2020), Gono et al. (2023)
Transcranial magnetic stimulation	PSD‐95	Postsynaptic density protein‐95	OGD/R injury in astrocytes	Increases axonal density and promotes neural plasticity	Hong et al. (2020)
	CaMKII	Calcium/Calmodulin‐dependent protein kinase‐II	OGD/R injury in astrocytes	Increases axonal density and promotes neural plasticity	Hong et al. (2020)
	Synapsin I	/	OGD/R injury in astrocytes	Increases axonal density and promotes neural plasticity	Hong et al. (2020)
	Synaptophysin	/	OGD/R injury in astrocytes	Increases axonal density and promotes neural plasticity	Hong et al. (2020)
	GDNF	Glial cell line‐derived neurotrophic factor	OGD injury in astrocytes	Contributes to in neuronal protection	Gava‐Junior et al. (2023), Roque et al. (2021)
	ERK1/2	Extracellular signal‐regulated kinase‐1/2	OGD/R in neuron‐glia cortical cultures	Associates with neuronal proliferation, differentiation, and apoptosis	Cruz‐Mendoza et al. (2022), Sahu et al. (2021)
	c‐Fos	/	OGD/R in neuron‐glia cortical cultures	A brain activity marker and attributes to neurons	Cruz‐Mendoza et al. (2022), Sahu et al. (2021)
	PDGFRβ	Platelet‐derived growth factor receptor beta	PT stroke model in rats	Associates with A2 astrocytes in stroke	Zong, Li, et al. (2020)
	TNF‐α	Tumor necrosis factor alpha	OGD/R in astrocytes	Proinflammatory cytokine and promotes neuronal death	Hong et al. (2020)
	IL‐10	Interleukin‐10	OGD/R in astrocytes	Anti‐inflammatory cytokine and alleviates neuronal death	Hong et al. (2020)
Transcranial electrical stimulation	SEPP1	Selenoprotein P1	Cerebral I/R injury in rats	Exerts a neural protect function	H. Wang et al. (2024)
Music therapy	BDNF	brain‐derived neurotrophic factor	MCAO model in rats	Promotes long‐term neuronal repair	W. Chen et al. (2021)
Acupuncture	BDNF	Brain‐derived neurotrophic factor	Cerebral ischemia‐reperfusion injury model in rats	Promotes neuroprotective effects	Tao et al. (2016)
	CB1R	Cannabinoid receptor type1	MCAO model in mice	Protects neurons from ischemic brain injury	C. Yang et al. (2021)
	MCT1	Monocarboxylate transporter 1	MCAO model in rats	Facilitates the transfer of lactate in the injured brain	Lu et al. (2015)
Hyperbaric oxygen therapy	NGF	Nerve growth factor	Cultured with 7% O_2_ in astrocytes	Promotes cell growth, survival and synaptic plasticity	Xing et al. (2018)
	BDNF	Brain‐derived neurotrophic factor	Cultured with 7% O_2_ in astrocytes	Promotes cell growth, survival and synaptic plasticity	Xing et al. (2018)
	NT‐3	Neurotrophin‐3	Cultured with 7% O_2_ in astrocytes	Promotes cell growth, survival and synaptic plasticity	Xing et al. (2018)
	GDNF	Glial cell line‐derived neurotrophic factor	Cultured with 7% O_2_ in astrocytes	Promotes cell growth, survival and synaptic plasticity	Xing et al. (2018)
Enriched environment	BDNF	Brain‐derived neurotrophic factor	MCAO model in rats	Promotes poststroke functional recovery	X. Chen et al. (2017), Guo and Bi (2024)
	VEGF	Vascular endothelial growth factor	MCAO model in rats	Promotes angiogenesis	Guo and Bi (2024)

This review summarizes the contributions of astrocytes to neuroprotection in various clinical rehabilitation settings. Activated astrocytes play essential roles in glial scarring, synaptic remodeling, inflammatory responses, glutamate transport, lactate metabolism, and BBB repair. These effects depend in part on various molecules secreted by reactive astrocytes, including NTFs, inflammatory factors, and neurotransmitters. Although targeting astrocyte autophagy may enhance neuroprotection in ischemic stroke, the modulation of astrocyte function involved in astrocyte autophagy has not been proposed.

Overall, we have provided an overview of the various ways in which astrocytes are important in stroke rehabilitation. Current data suggest that astrocytes are promising targets for therapeutic intervention in future rehabilitation research. However, the modulation of astrocyte function in ischemic stroke remains controversial. Therefore, further investigation is needed before attempting to utilize astrocytes for rehabilitative applications.

## Author Contributions

**Ying Han**: conceptualization, investigation, funding acquisition, writing – original draft, writing – review and editing, visualization, formal analysis, project administration. **Li Huang**: conceptualization, investigation, writing – review and editing. **Jiaojiao Wu**: investigation. **Guiling Wan**: investigation. **Linhong Mo**: supervision, data curation, project administration, writing – review and editing. **Aixian Liu**: data curation, supervision, resources, project administration, writing – review and editing.

## Conflicts of Interest

The authors declare no conflicts of interest.

### Peer Review

The peer review history for this article is available at https://publons.com/publon/10.1002/brb3.70551.

## Data Availability

Data sharing does not apply to this article, as no new data were created or analyzed in this study.

## References

[brb370551-bib-0001] Ahn, J. H. , M. C. Shin , J. H. Park , et al. 2017. “Effects of Long‑Term Post‑Ischemic Treadmill Exercise on Gliosis in the Aged Gerbil Hippocampus Induced by Transient Cerebral Ischemia.” Molecular Medicine Reports 15, no. 6: 3623–3630. 10.3892/mmr.2017.6485. 28440411 PMC5436201

[brb370551-bib-0002] Baba, H. , K. Nakahira , N. Morita , F. Tanaka , H. Akita , and K. Ikenaka . 1997. “GFAP Gene Expression During Development of Astrocyte.” Developmental Neuroscience 19, no. 1: 49–57. 10.1159/000111185. 9078433

[brb370551-bib-0003] Barthels, D. , and H. Das . 2020. “Current Advances in Ischemic Stroke Research and Therapies.” Biochimica et Biophysica Acta (BBA)‐Molecular Basis of Disease 1866, no. 4: 165260. 10.1016/j.bbadis.2018.09.012. 31699365 PMC6981280

[brb370551-bib-0004] Belaya, I. , M. Ivanova , A. Sorvari , et al. 2020. “Astrocyte Remodeling in the Beneficial Effects of Long‐Term Voluntary Exercise in Alzheimer's Disease.” Journal of Neuroinflammation 17, no. 1: 271. 10.1186/s12974-020-01935-w. 32933545 PMC7493971

[brb370551-bib-0005] Benz, F. , and S. Liebner . 2022. “Structure and Function of the Blood‐Brain Barrier (BBB).” Handbook of Experimental Pharmacology 273: 3–31. 10.1007/164_2020_404. 33249527

[brb370551-bib-0006] Bonvento, G. , and J. P. Bolaños . 2021. “Astrocyte‐Neuron Metabolic Cooperation Shapes Brain Activity.” Cell Metabolism 33, no. 8: 1546–1564. 10.1016/j.cmet.2021.07.006. 34348099

[brb370551-bib-0007] Brann, D. W. , Y. Lu , J. Wang , et al. 2022. “Brain‐Derived Estrogen and Neural Function.” Neuroscience and Biobehavioral Reviews 132: 793–817. 10.1016/j.neubiorev.2021.11.014. 34823913 PMC8816863

[brb370551-bib-0008] Caglayan, A. B. , M. C. Beker , E. Sertel Evren , et al. 2023. “The Unconventional Growth Factors Cerebral Dopamine Neurotrophic Factor and Mesencephalic Astrocyte‐Derived Neurotrophic Factor Promote Post‐Ischemic Neurological Recovery, Perilesional Brain Remodeling, and Lesion‐Remote Axonal Plasticity.” Translational Stroke Research 14, no. 2: 263–277. 10.1007/s12975-022-01035-2. 35583716

[brb370551-bib-0009] Caliaperumal, J. , and F. Colbourne . 2014. “Rehabilitation Improves Behavioral Recovery and Lessens Cell Death Without Affecting Iron, Ferritin, Transferrin, or Inflammation After Intracerebral Hemorrhage in Rats.” Neurorehabilitation and Neural Repair 28, no. 4: 395–404. 10.1177/1545968313517758. 24376068

[brb370551-bib-0010] Campbell, B. C. V. , D. A. De Silva , M. R. Macleod , et al. 2019. “Ischaemic Stroke.” Nature Reviews Disease Primers 5, no. 1: 70. 10.1038/s41572-019-0118-8. 31601801

[brb370551-bib-0011] Chan, P. , L. F. Eng , Y. Ling Lee , and V. W. H. Lin . 1999. “Effects of Pulsed Magnetic Stimulation of GFAP Levels in Cultured Astrocytes.” Journal of Neuroscience Research 55, no. 2: 238–244. 10.1002/(sici)1097-4547(19990115)55:2<238::aid-jnr11>3.0.co;2-t. 9972826

[brb370551-bib-0012] Chen, W. , J. Zheng , G. Shen , et al. 2021. “Music Therapy Alleviates Motor Dysfunction in Rats With Focal Cerebral Ischemia‐Reperfusion Injury by Regulating BDNF Expression.” Frontiers in Neurology 12: 666311. 10.3389/fneur.2021.666311. 34262520 PMC8273236

[brb370551-bib-0013] Chen, X. , X. Zhang , W. Liao , and Q. Wan . 2017. “Effect of Physical and Social Components of Enriched Environment on Astrocytes Proliferation in Rats After Cerebral Ischemia/Reperfusion Injury.” Neurochemical Research 42, no. 5: 1308–1316. 10.1007/s11064-016-2172-x. 28083848

[brb370551-bib-0014] Chen, Y. L. , D. Li , Z. Z. Wang , W. G. Xu , R. P. Li , and J. D. Zhang . 2016. “Glutamate Metabolism of Astrocytes During Hyperbaric Oxygen Exposure and Its Effects on Central Nervous System Oxygen Toxicity.” Neuroreport 27, no. 2: 73–79. 10.1097/wnr.0000000000000493. 26619231

[brb370551-bib-0015] Chen, Y. L. , Y. N. Zhang , Z. Z. Wang , W. G. Xu , R. P. Li , and J. D. Zhang . 2016. “Effects of Adenosine Metabolism in Astrocytes on Central Nervous System Oxygen Toxicity.” Brain Research 1635: 180–189. 10.1016/j.brainres.2016.01.026. 26806404

[brb370551-bib-0016] Cho, W.‐H. , K. Noh , B. H. Lee , et al. 2022. “Hippocampal Astrocytes Modulate Anxiety‐Like Behavior.” Nature Communications 13, no. 1: 6536. 10.1038/s41467-022-34201-z. PMC9640657 36344520

[brb370551-bib-0017] Clain, J. , D. Couret , M. Bringart , et al. 2024. “Metabolic Disorders Exacerbate the Formation of Glial Scar After Stroke.” European Journal of Neuroscience 59, no. 11: 3009–3029. 10.1111/ejn.16325. 38576159

[brb370551-bib-0018] Clarke, D. , M. A. Penrose , T. Penstone , et al. 2017. “Frequency‐Specific Effects of Repetitive Magnetic Stimulation on Primary Astrocyte Cultures.” Restorative Neurology and Neuroscience 35, no. 6: 557–569. 10.3233/rnn-160708. 29172007

[brb370551-bib-0019] Collimore, A. N. , A. V. Roto Cataldo , A. J. Aiello , et al. 2023. “Autonomous Control of Music to Retrain Walking after Stroke.” Neurorehabilitation and Neural Repair 37, no. 5: 255–265. 10.1177/15459683231174223. 37272500 PMC10272623

[brb370551-bib-0020] Cruz‐Mendoza, F. , F. Jauregui‐Huerta , A. Aguilar‐Delgadillo , J. García‐Estrada , and S. Luquin . 2022. “Immediate Early Gene c‐fos in the Brain: Focus on Glial Cells.” Brain Sciences 12, no. 6: 687. 10.3390/brainsci12060687. 35741573 PMC9221432

[brb370551-bib-0021] Dai, W.‐J. , J.‐L. Sun , C. Li , et al. 2019. “Involvement of Interleukin‐10 in Analgesia of Electroacupuncture on Incision Pain.” Evidence‐Based Complementary and Alternative Medicine 2019: 1–11. 10.1155/2019/8413576. PMC6925708 31885668

[brb370551-bib-0022] De Wolde, S. D. , R. H. Hulskes , R. P. Weenink , M. W. Hollmann , and R. A. Van Hulst . 2021. “The Effects of Hyperbaric Oxygenation on Oxidative Stress, Inflammation and Angiogenesis.” Biomolecules 11, no. 8: 1210. 10.3390/biom11081210. 34439876 PMC8394403

[brb370551-bib-0023] Ding, Y. , P. Yao , T. Hong , et al. 2018. “The Analgesic Effect of Early Hyperbaric Oxygen Treatment in Chronic Constriction Injury Rats and Its Influence on nNOS and iNOS Expression and Inflammatory Factor Production.” Molecular Pain 14: 1744806918765837. 10.1177/1744806918765837. 29592784 PMC5881973

[brb370551-bib-0024] Ding, Y. H. , Y. Ding , J. Li , D. A. Bessert , and J. A. Rafols . 2006. “Exercise Pre‐Conditioning Strengthens Brain Microvascular Integrity in a Rat Stroke Model.” Neurological Research 28, no. 2: 184–189. 10.1179/016164106x98053. 16551437

[brb370551-bib-0025] Ding, Z. B. , L. J. Song , Q. Wang , G. Kumar , Y. Q. Yan , and C. G. Ma . 2021. “Astrocytes: A Double‐Edged Sword in Neurodegenerative Diseases.” Neural Regeneration Research 16, no. 9: 1702–1710. 10.4103/1673-5374.306064. 33510058 PMC8328766

[brb370551-bib-0026] Di Raimondo, D. , G. Rizzo , G. Musiari , A. Tuttolomondo , and A. Pinto . 2020. “Role of Regular Physical Activity in Neuroprotection Against Acute Ischemia.” International Journal of Molecular Sciences 21, no. 23: 9086. 10.3390/ijms21239086. 33260365 PMC7731306

[brb370551-bib-0027] Durkee, C. A. , and A. Araque . 2019. “Diversity and Specificity of Astrocyte‐Neuron Communication.” Neuroscience 396: 73–78. 10.1016/j.neuroscience.2018.11.010. 30458223 PMC6494094

[brb370551-bib-0028] Dutra, M. F. , M. Jaeger , J. Ilha , P. I. Kalil‐Gaspar , S. Marcuzzo , and M. Achaval . 2012. “Exercise Improves Motor Deficits and Alters Striatal GFAP Expression in a 6‐OHDA‐Induced Rat Model of Parkinson's Disease.” Neurological Sciences 33, no. 5: 1137–1144. 10.1007/s10072-011-0925-5. 22231471

[brb370551-bib-0029] Eckert, M. J. , and W. C. Abraham . 2013. “Effects of Environmental Enrichment Exposure on Synaptic Transmission and Plasticity in the Hippocampus.” Current Topics in Behavioral Neurosciences 15: 165–187. 10.1007/7854_2012_215. 22798066

[brb370551-bib-0030] Escartin, C. , E. Galea , A. Lakatos , et al. 2021. “Reactive Astrocyte Nomenclature, Definitions, and Future Directions.” Nature Neuroscience 24, no. 3: 312–325. 10.1038/s41593-020-00783-4. 33589835 PMC8007081

[brb370551-bib-0031] Fei, X. , Y.‐N. Dou , L. Wang , et al. 2022. “Homer1 promotes the Conversion of A1 Astrocytes to A2 Astrocytes and Improves the Recovery of Transgenic Mice After Intracerebral Hemorrhage.” Journal of Neuroinflammation 19, no. 1: 67. 10.1186/s12974-022-02428-8. 35287697 PMC8922810

[brb370551-bib-0032] Filho, P. R. M. , R. Vercelino , S. G. Cioato , et al. 2016. “Transcranial Direct Current Stimulation (tDCS) Reverts Behavioral Alterations and Brainstem BDNF Level Increase Induced by Neuropathic Pain Model: Long‐Lasting Effect.” Progress in Neuro‐Psychopharmacology & Biological Psychiatry 64: 44–51. 10.1016/j.pnpbp.2015.06.016. 26160698

[brb370551-bib-0033] François, C. , J. Grau‐Sánchez , E. Duarte , and A. Rodriguez‐Fornells . 2015. “Musical Training as an Alternative and Effective Method for Neuro‐Education and Neuro‐Rehabilitation.” Frontiers in Psychology 6: 475. 10.3389/fpsyg.2015.00475. 25972820 PMC4411999

[brb370551-bib-0034] Gao, Z. K. , X. Y. Shen , Y. Han , Y. S. Guo , M. Yuan , and X. Bi . 2022. “Enriched Environment Effects on Myelination of the Central Nervous System: Role of Glial Cells.” Neural Plasticity 2022: 1–16. 10.1155/2022/5766993. PMC9023233 35465398

[brb370551-bib-0035] Gardner‐Medwin, A. R. , and C. Nicholson . 1983. “Changes of Extracellular Potassium Activity Induced by Electric Current Through Brain Tissue in the Rat.” Journal of Physiology 335: 375–392. 10.1113/jphysiol.1983.sp014540. 6875884 PMC1197359

[brb370551-bib-0036] Gava‐Junior, G. , S. A. Ferreira , C. Roque , et al. 2023. “High‐Frequency Repetitive Magnetic Stimulation Rescues Ischemia‐injured Neurons Through Modulation of Glial‐Derived Neurotrophic Factor Present in the Astrocyte's Secretome.” Journal of Neurochemistry 164, no. 6: 813–828. 10.1111/jnc.15740. 36477745

[brb370551-bib-0037] Gim, G.‐T. , J.‐H. Lee , E. Park , et al. 2011. “Electroacupuncture Attenuates Mechanical and Warm Allodynia Through Suppression of Spinal Glial Activation in a Rat Model of Neuropathic Pain.” Brain Research Bulletin 86, no. 5–6: 403–411. 10.1016/j.brainresbull.2011.09.010. 21958939

[brb370551-bib-0038] Gono, R. , K. Sugimoto , C. Yang , et al. 2023. “Molecular Mechanism of Cerebral Edema Improvement via IL‐1RA Released From the Stroke‐Unaffected Hindlimb by Treadmill Exercise After Cerebral Infarction in Rats.” Journal of Cerebral Blood Flow and Metabolism 43, no. 5: 812–827. 10.1177/0271678x231151569. 36651110 PMC10108195

[brb370551-bib-0039] Grau‐Sánchez, J. , J. L. Amengual , N. Rojo , et al. 2013. “Plasticity in the Sensorimotor Cortex Induced by Music‐Supported Therapy in Stroke Patients: A TMS Study.” Frontiers in Human Neuroscience 7: 494. 10.3389/fnhum.2013.00494. 24027507 PMC3759754

[brb370551-bib-0040] Grau‐Sánchez, J. , E. Duarte , N. Ramos‐Escobar , et al. 2018. “Music‐Supported Therapy in the Rehabilitation of Subacute Stroke Patients: A Randomized Controlled Trial.” Annals of the New York Academy of Sciences 1423: 318–328. 10.1111/nyas.13590. 29607506

[brb370551-bib-0041] Günther, A. , L. Küppers‐Tiedt , P.‐M. Schneider , et al. 2005. “Reduced Infarct Volume and Differential Effects on Glial Cell Activation After Hyperbaric Oxygen Treatment in Rat Permanent Focal Cerebral Ischaemia.” European Journal of Neuroscience 21, no. 11: 3189–3194. 10.1111/j.1460-9568.2005.04151.x. 15978027

[brb370551-bib-0042] Guo, Y. S. , and X. Bi . 2024. “Enriched Environment Enhanced the Astrocyte‐Derived BDNF and VEGF Expression and Alleviate White Matter Injuries of Rats With Ischemic Stroke.” Neurological Research 46, no. 3: 272–283. 10.1080/01616412.2023.2298136. 38145566

[brb370551-bib-0043] Han, P. P. , Y. Han , X. Y. Shen , Z. K. Gao , and X. Bi . 2023. “Enriched Environment‐Induced Neuroplasticity in Ischemic Stroke and Its Underlying Mechanisms.” Frontiers in Cellular Neuroscience 17: 1210361. 10.3389/fncel.2023.1210361. 37484824 PMC10360187

[brb370551-bib-0044] Hol, E. M. , and M. Pekny . 2015. “Glial Fibrillary Acidic Protein (GFAP) and the Astrocyte Intermediate Filament System in Diseases of the Central Nervous System.” Current Opinion in Cell Biology 32: 121–130. 10.1016/j.ceb.2015.02.004. 25726916

[brb370551-bib-0045] Hong, Y. , Q. Liu , M. Peng , et al. 2020. “High‐Frequency Repetitive Transcranial Magnetic Stimulation Improves Functional Recovery by Inhibiting Neurotoxic Polarization of Astrocytes in Ischemic Rats.” Journal of Neuroinflammation 17, no. 1: 150. 10.1186/s12974-020-01747-y. 32375835 PMC7203826

[brb370551-bib-0046] Johnson, K. M. , R. Milner , and S. J. Crocker . 2015. “Extracellular Matrix Composition Determines Astrocyte Responses to Mechanical and Inflammatory Stimuli.” Neuroscience Letters 600: 104–109. 10.1016/j.neulet.2015.06.013. 26067407 PMC4530625

[brb370551-bib-0047] Jones, T. A. , and W. T. Greenough . 1996. “Ultrastructural Evidence for Increased Contact Between Astrocytes and Synapses in Rats Reared in a Complex Environment.” Neurobiology of Learning and Memory 65, no. 1: 48–56. 10.1006/nlme.1996.0005. 8673406

[brb370551-bib-0048] Jurič, D. M. , D. Lončar , and M. Čarman‐Kržan . 2008. “Noradrenergic Stimulation of BDNF Synthesis in Astrocytes: Mediation via Alpha1‐ and Beta1/Beta2‐Adrenergic Receptors.” Neurochemistry International 52, no. 1–2: 297–306. 10.1016/j.neuint.2007.06.035. 17681645

[brb370551-bib-0049] Justicia, C. , C. Gabriel , and A. M. Planas . 2000. “Activation of the JAK/STAT Pathway Following Transient Focal Cerebral Ischemia: Signaling Through Jak1 and Stat3 in Astrocytes.” Glia 30, no. 3: 253–270. 10.1002/(sici)1098-1136(200005)30:3<253::aid-glia5>3.0.co;2-o. 10756075

[brb370551-bib-0050] Kempermann, G. 2019. “Environmental Enrichment, New Neurons and the Neurobiology of Individuality.” Nature Reviews Neuroscience 20, no. 4: 235–245. 10.1038/s41583-019-0120-x. 30723309

[brb370551-bib-0051] Kiper, P. , E. Przysiężna , B.ż Cieślik , et al. 2022. “Effects of Immersive Virtual Therapy as a Method Supporting Recovery of Depressive Symptoms in Post‐Stroke Rehabilitation: Randomized Controlled Trial.” CIA 17: 1673–1685. 10.2147/cia.s375754. PMC9701456 36447623

[brb370551-bib-0052] Li, F. , X. Geng , H. J. Yun , Y. Haddad , Y. Chen , and Y. Ding . 2021. “Neuroplastic Effect of Exercise Through Astrocytes Activation and Cellular Crosstalk.” Aging and Disease 12, no. 7: 1644–1657. 10.14336/ad.2021.0325. 34631212 PMC8460294

[brb370551-bib-0053] Liang, Y. , Y. Qiu , J. Du , et al. 2016. “Inhibition of Spinal Microglia and Astrocytes Contributes to the Anti‐Allodynic Effect of Electroacupuncture in Neuropathic Pain Induced by Spinal Nerve Ligation.” Acupuncture in Medicine 34, no. 1: 40–47. 10.1136/acupmed-2015-010773. 26177687

[brb370551-bib-0054] Lin, C. M. , C. K. Chang , C. P. Chang , Y. C. Hsu , M. T. Lin , and J. W. Lin . 2015. “Protecting Against Ischaemic Stroke in Rats by Heat Shock Protein 20‐Mediated Exercise.” European Journal of Clinical Investigation 45, no. 12: 1297–1305. 10.1111/eci.12551. 26479875

[brb370551-bib-0055] Lippert, T. , and C. V. Borlongan . 2019. “Prophylactic Treatment of Hyperbaric Oxygen Treatment Mitigates Inflammatory Response via Mitochondria Transfer.” CNS neuroscience & therapeutics 25, no. 8: 815–823. 10.1111/cns.13124. 30972972 PMC6630002

[brb370551-bib-0056] Liu, Y. X. , H. Sun , and W. Y. Guo . 2022. “Astrocyte Polarization in Glaucoma: A New Opportunity.” Neural Regeneration Research 17, no. 12: 2582–2588. 10.4103/1673-5374.339470. 35662185 PMC9165360

[brb370551-bib-0057] Liu, Z. , Y. Li , Y. Cui , et al. 2014. “Beneficial Effects of gfap/Vimentin Reactive Astrocytes for Axonal Remodeling and Motor Behavioral Recovery in Mice After Stroke.” Glia 62, no. 12: 2022–2033. 10.1002/glia.22723. 25043249 PMC4307923

[brb370551-bib-0058] Lu, Y. , H. Zhao , Y. Wang , et al. 2015. “Electro‐Acupuncture Up‐Regulates Astrocytic MCT1 Expression to Improve Neurological Deficit in Middle Cerebral Artery Occlusion Rats.” Life Sciences 134: 68–72. 10.1016/j.lfs.2015.05.014. 26037401

[brb370551-bib-0059] Magistretti, P. J. 2006. “Neuron‐Glia Metabolic Coupling and Plasticity.” Journal of Experimental Biology 209, no. Pt 12: 2304–2311. 10.1242/jeb.02208. 16731806

[brb370551-bib-0060] Markham, J. A. , and W. T. Greenough . 2004. “Experience‐driven Brain Plasticity: Beyond the Synapse.” Neuron Glia Biology 1, no. 4: 351–363. 10.1017/s1740925x05000219. 16921405 PMC1550735

[brb370551-bib-0061] Monai, H. , and H. Hirase . 2016. “Astrocytic Calcium Activation in a Mouse Model of tDCS‐Extended Discussion.” Neurogenesis (Austin) 3, no. 1: e1240055. 10.1080/23262133.2016.1240055. 27830161 PMC5079391

[brb370551-bib-0062] Monai, H. , M. Ohkura , M. Tanaka , et al. 2016. “Calcium Imaging Reveals Glial Involvement in Transcranial Direct Current Stimulation‐Induced Plasticity in Mouse Brain.” Nature Communications 7: 11100. 10.1038/ncomms11100. PMC4804173 27000523

[brb370551-bib-0063] Murata, Y. , K. Sugimoto , C. Yang , et al. 2020. “Activated Microglia‐Derived Macrophage‐Like Cells Exacerbate Brain Edema After Ischemic Stroke Correlate With Astrocytic Expression of Aquaporin‐4 and Interleukin‐1 Alpha Release.” Neurochemistry International 140: 104848. 10.1016/j.neuint.2020.104848. 32920036

[brb370551-bib-0064] Oosthuyse, T. , J. A. Strauss , and A. C. Hackney . 2023. “Understanding the Female Athlete: Molecular Mechanisms Underpinning Menstrual Phase Differences in Exercise Metabolism.” European Journal of Applied Physiology 123, no. 3: 423–450. 10.1007/s00421-022-05090-3. 36402915

[brb370551-bib-0065] Parabucki, A. B. , I. D. Božić , I. M. Bjelobaba , et al. 2012. “Hyperbaric Oxygenation Alters Temporal Expression Pattern of Superoxide Dismutase 2 After Cortical Stab Injury in Rats.” Croatian Medical Journal 53, no. 6: 586–597. 10.3325/cmj.2012.53.586. 23275324 PMC3547292

[brb370551-bib-0066] Qian, J. , X. Ren , X. Wang , et al. 2009. “Blockade of Hsp20 Phosphorylation Exacerbates Cardiac Ischemia/Reperfusion Injury by Suppressed Autophagy and Increased Cell Death.” Circulation Research 105, no. 12: 1223–1231. 10.1161/circresaha.109.200378. 19850943 PMC2799045

[brb370551-bib-0067] Raglio, A. 2015. “Effects of Music and Music Therapy on Mood in Neurological Patients.” World Journal of Psychiatry 5, no. 1: 68–78. 10.5498/wjp.v5.i1.68. 25815256 PMC4369551

[brb370551-bib-0068] Raglio, A. , A. Zaliani , P. Baiardi , et al. 2017. “Active Music Therapy Approach for Stroke Patients in the Post‐Acute Rehabilitation.” Neurological Sciences 38, no. 5: 893–897. 10.1007/s10072-017-2827-7. 28138867

[brb370551-bib-0069] Rao, Y. , J. Li , R. Qiao , J. Luo , and Y. Liu . 2024. “Synergistic Effects of Tetramethylpyrazine and Astragaloside IV on Spinal Cord Injury via Alteration of Astrocyte A1/A2 Polarization Through the Sirt1‐NF‐κB Pathway.” International Immunopharmacology 131: 111686. 10.1016/j.intimp.2024.111686. 38461631

[brb370551-bib-0070] Rocha, S. M. , A. C. Cristovão , F. L. Campos , C. P. Fonseca , and G. Baltazar . 2012. “Astrocyte‐Derived GDNF Is a Potent Inhibitor of Microglial Activation.” Neurobiology of Disease 47, no. 3: 407–415. 10.1016/j.nbd.2012.04.014. 22579772

[brb370551-bib-0071] Rodriguez‐Fornells, A. , N. Rojo , J. L. Amengual , P. Ripollés , E. Altenmüller , and T. F. Münte . 2012. “The Involvement of Audio‐Motor Coupling in the Music‐Supported Therapy Applied to Stroke Patients.” Annals of the New York Academy of Sciences 1252: 282–293. 10.1111/j.1749-6632.2011.06425.x. 22524370

[brb370551-bib-0072] Roque, C. , N. Pinto , M. Vaz Patto , and G. Baltazar . 2021. “Astrocytes Contribute to the Neuronal Recovery Promoted by High‐Frequency Repetitive Magnetic Stimulation in In Vitro Models of Ischemia.” Journal of Neuroscience Research 99, no. 5: 1414–1432. 10.1002/jnr.24792. 33522025

[brb370551-bib-0073] Ruohonen, J. , and J. Karhu . 2012. “tDCS Possibly Stimulates Glial Cells.” Clinical Neurophysiology 123, no. 10: 2006–2009. 10.1016/j.clinph.2012.02.082. 22480602

[brb370551-bib-0074] Sahu, R. , S. Upadhayay , and S. Mehan . 2021. “Inhibition of Extracellular Regulated Kinase (ERK)‐1/2 Signaling Pathway in the Prevention of ALS: Target Inhibitors and Influences on Neurological Dysfunctions.” European Journal of Cell Biology 100, no. 7–8: 151179. 10.1016/j.ejcb.2021.151179. 34560374

[brb370551-bib-0075] Saidi, M. , and S. M. Firoozabadi . 2021. “Glial Cells Have More Important Role in tDCS‐Induced Brain Activities Rather Than Neurons.” Medical Hypotheses 153: 110615. 10.1016/j.mehy.2021.110615. 34214759

[brb370551-bib-0076] Saur, L. , P. P. A. Baptista , P. N. De Senna , et al. 2014. “Physical Exercise Increases GFAP Expression and Induces Morphological Changes in Hippocampal Astrocytes.” Brain Structure and Function 219, no. 1: 293–302. 10.1007/s00429-012-0500-8. 23288255

[brb370551-bib-0077] Seifert, G. , C. Henneberger , and C. Steinhäuser . 2018. “Diversity of Astrocyte Potassium Channels: An Update.” Brain Research Bulletin 136: 26–36. 10.1016/j.brainresbull.2016.12.002. 27965079

[brb370551-bib-0078] Sihvonen, A. J. , V. Leo , P. Ripollés , et al. 2020. “Vocal Music Enhances Memory and Language Recovery After Stroke: Pooled Results From Two RCTs.” Annals of Clinical and Translational Neurology 7, no. 11: 2272–2287. 10.1002/acn3.51217. 33022148 PMC7664275

[brb370551-bib-0079] Sihvonen, A. J. , P. Ripollés , V. Leo , et al. 2021. “Vocal Music Listening Enhances Post‐Stroke Language Network Reorganization.” eNeuro 8, no. 4: ENEURO.0158–0121.2021. 10.1523/eneuro.0158-21.2021. 34140351 PMC8266215

[brb370551-bib-0080] Sihvonen, A. J. , S. Soinila , and T. Särkämö . 2022. “Post‐Stroke Enriched Auditory Environment Induces Structural Connectome Plasticity: Secondary Analysis From a Randomized Controlled Trial.” Brain Imaging and Behavior 16, no. 4: 1813–1822. 10.1007/s11682-022-00661-6. 35352235 PMC9279272

[brb370551-bib-0081] Silver, J. , and J. H. Miller . 2004. “Regeneration Beyond the Glial Scar.” Nature Reviews Neuroscience 5, no. 2: 146–156. 10.1038/nrn1326. 14735117

[brb370551-bib-0082] Song, S. , H. Huang , X. Guan , et al. 2021. “Activation of Endothelial Wnt/β‐Catenin Signaling by Protective Astrocytes Repairs BBB Damage in Ischemic Stroke.” Progress in Neurobiology 199: 101963. 10.1016/j.pneurobio.2020.101963. 33249091 PMC7925353

[brb370551-bib-0083] Stogsdill, J. A. , J. Ramirez , D. Liu , et al. 2017. “Astrocytic Neuroligins Control Astrocyte Morphogenesis and Synaptogenesis.” Nature 551, no. 7679: 192–197. 10.1038/nature24638. 29120426 PMC5796651

[brb370551-bib-0084] Sun, J. , X. Zhou , B. Ren , et al. 2024. “Effects of Acupuncture Combined With Five‐Element Music for People With Mild/Moderate Post‐Stroke Depression: A Randomized Controlled Trial.” Complementary Therapies in Medicine 86: 103088. 10.1016/j.ctim.2024.103088. 39332596

[brb370551-bib-0085] Tao, J. , Y. Zheng , W. Liu , et al. 2016. “Electro‐Acupuncture at LI11 and ST36 Acupoints Exerts Neuroprotective Effects via Reactive Astrocyte Proliferation After Ischemia and Reperfusion Injury in Rats.” Brain Research Bulletin 120: 14–24. 10.1016/j.brainresbull.2015.10.011. 26524137

[brb370551-bib-0086] Tuo, Q. Z. , S. T. Zhang , and P. Lei . 2022. “Mechanisms of Neuronal Cell Death in Ischemic Stroke and Their Therapeutic Implications.” Medicinal Research Reviews 42, no. 1: 259–305. 10.1002/med.21817. 33957000

[brb370551-bib-0087] Wang, H. , W. Ma , W. Hu , et al. 2024. “Cathodal Bilateral Transcranial Direct‐Current Stimulation Regulates Selenium to Confer Neuroprotection After Rat Cerebral Ischaemia‐Reperfusion Injury.” Journal of Physiology 602, no. 6: 1175–1197. 10.1113/jp285806. 38431908

[brb370551-bib-0088] Wang, J. , G. R. Sareddy , Y. Lu , et al. 2020. “Astrocyte‐Derived Estrogen Regulates Reactive Astrogliosis and Is Neuroprotective Following Ischemic Brain Injury.” Journal of Neuroscience 40, no. 50: 9751–9771. 10.1523/jneurosci.0888-20.2020. 33158962 PMC7726540

[brb370551-bib-0089] Wang, J.‐Y. , Y.‐H. Gao , L.‐N. Qiao , et al. 2018. “Repeated Electroacupuncture Treatment Attenuated Hyperalgesia Through Suppression of Spinal Glial Activation in Chronic Neuropathic Pain Rats.” BMC Complementary and Alternative Medicine [Electronic Resource] 18, no. 1: 74. 10.1186/s12906-018-2134-8. 29466978 PMC5822602

[brb370551-bib-0090] Wang, L. C. , W. Y. Wei , P. C. Ho , P. Y. Wu , Y. P. Chu , and K. J. Tsai . 2021. “Somatosensory Cortical Electrical Stimulation After Reperfusion Attenuates Ischemia/Reperfusion Injury of Rat Brain.” Frontiers in Aging Neuroscience 13: 741168. 10.3389/fnagi.2021.741168. 34867274 PMC8632773

[brb370551-bib-0091] Wang, Y. , and H. Monai . 2024. “Transcranial Direct Current Stimulation Alters Cerebrospinal Fluid‐Interstitial Fluid Exchange in Mouse Brain.” Brain Stimulation 17, no. 3: 620–632. 10.1016/j.brs.2024.04.009. 38688399

[brb370551-bib-0092] Wang, Y. , W.‐Y. Pan , F. Li , et al. 2021. “Effect of Rhythm of Music Therapy on Gait in Patients With Stroke.” Journal of Stroke and Cerebrovascular Diseases 30, no. 3: 105544. 10.1016/j.jstrokecerebrovasdis.2020.105544. 33341022

[brb370551-bib-0093] Xia, A. , H. Huang , W. You , Y. Liu , H. Wu , and S. Liu . 2022. “The Neuroprotection of Hyperbaric Oxygen Therapy Against Traumatic Brain Injury via NF‐κB/MAPKs‐CXCL1 Signaling Pathways.” Experimental Brain Research 240, no. 1: 207–220. 10.1007/s00221-021-06249-8. 34687331

[brb370551-bib-0094] Xiao, J. , T. Wang , B. Ye , and C. Tang . 2023. “Scalp Acupuncture and Computer Assisted Cognitive Rehabilitation for Stroke: A Meta‐Analysis of Randomised Controlled Trials.” Heliyon 9, no. 7: e18157. 10.1016/j.heliyon.2023.e18157. 37501979 PMC10368847

[brb370551-bib-0095] Xing, P. , K. Ma , L. Li , D. Wang , G. Hu , and W. Long . 2018. “The Protection Effect and Mechanism of Hyperbaric Oxygen Therapy in Rat Brain With Traumatic Injury.” Acta Cirurgica Brasileira 33, no. 4: 341–353. 10.1590/s0102-865020180040000006. 29768537

[brb370551-bib-0096] Xu, J. , G. Huang , K. Zhang , et al. 2014. “Nrf2 Activation in Astrocytes Contributes to Spinal Cord Ischemic Tolerance Induced by Hyperbaric Oxygen Preconditioning.” Journal of Neurotrauma 31, no. 15: 1343–1353. 10.1089/neu.2013.3222. 24716787 PMC4120927

[brb370551-bib-0097] Xu, X. , A. Zhang , Y. Zhu , et al. 2018. “MFG‐E8 Reverses Microglial‐Induced Neurotoxic Astrocyte (A1) via NF‐κB and PI3K‐Akt Pathways.” Journal of Cellular Physiology 234, no. 1: 904–914. 10.1002/jcp.26918. 30076715

[brb370551-bib-0098] Yamagata, K. 2022. “Lactate Supply From Astrocytes to Neurons and Its Role in Ischemic Stroke‐Induced Neurodegeneration.” Neuroscience 481: 219–231. 10.1016/j.neuroscience.2021.11.035. 34843897

[brb370551-bib-0099] Yang, C. , J. Liu , J. Wang , et al. 2021. “Activation of Astroglial CB1R Mediates Cerebral Ischemic Tolerance Induced by Electroacupuncture.” Journal of Cerebral Blood Flow and Metabolism 41, no. 9: 2295–2310. 10.1177/0271678x21994395. 33663269 PMC8393297

[brb370551-bib-0100] Yang, X. , Z. He , Q. Zhang , et al. 2012. “Pre‐Ischemic Treadmill Training for Prevention of Ischemic Brain Injury via Regulation of Glutamate and Its Transporter GLT‐1.” International Journal of Molecular Sciences 13, no. 8: 9447–9459. 10.3390/ijms13089447. 22949807 PMC3431805

[brb370551-bib-0101] Yang, Y. , S. Vidensky , L. Jin , et al. 2011. “Molecular Comparison of GLT1+ and ALDH1L1+ Astrocytes In Vivo in Astroglial Reporter Mice.” Glia 59, no. 2: 200–207. 10.1002/glia.21089. 21046559 PMC3199134

[brb370551-bib-0102] Yeh, T. H. , H. M. Hwang , J. J. Chen , T. Wu , A. H. Li , and H. L. Wang . 2005. “Glutamate Transporter Function of Rat Hippocampal Astrocytes Is Impaired Following the Global Ischemia.” Neurobiology of Disease 18, no. 3: 476–483. 10.1016/j.nbd.2004.12.011. 15755674

[brb370551-bib-0103] Zhang, F. , J. Jia , Y. Wu , Y. Hu , and Y. Wang . 2010. “The Effect of Treadmill Training Pre‐Exercise on Glutamate Receptor Expression in Rats After Cerebral Ischemia.” International Journal of Molecular Sciences 11, no. 7: 2658–2669. 10.3390/ijms11072658. 20717528 PMC2920558

[brb370551-bib-0104] Zhang, K.‐Y. , G. Rui , J.‐P. Zhang , et al. 2020. “Cathodal tDCS Exerts Neuroprotective Effect in Rat Brain After Acute Ischemic Stroke.” BMC Neuroscience [Electronic Resource] 21, no. 1: 21. 10.1186/s12868-020-00570-8. 32397959 PMC7216334

[brb370551-bib-0105] Zhang, Q. , and P. G. Haydon . 2005. “Roles for Gliotransmission in the Nervous System.” Journal of Neural Transmission (Vienna) 112, no. 1: 121–125. 10.1007/s00702-004-0119-x. 15599610

[brb370551-bib-0106] Zhang, R. , Y. Wu , F. Xie , et al. 2018. “RGMa Mediates Reactive Astrogliosis and Glial Scar Formation Through TGFβ1/Smad2/3 Signaling After Stroke.” Cell Death and Differentiation 25, no. 8: 1503–1516. 10.1038/s41418-018-0058-y. 29396549 PMC6113216

[brb370551-bib-0107] Zhang, T. , W. Deng , Y. Deng , et al. 2023. “Mechanisms of Ferroptosis Regulating Oxidative Stress and Energy Metabolism in Myocardial Ischemia‐Reperfusion Injury and a Novel Perspective of Natural Plant Active Ingredients for Its Treatment.” Biomedicine & Pharmacotherapy 165: 114706. 10.1016/j.biopha.2023.114706. 37400352

[brb370551-bib-0108] Zhang, X. , Z. Talifu , J. Li , X. Li , and F. Yu . 2023. “Melodic Intonation Therapy for Non‐Fluent Aphasia After Stroke: A Clinical Pilot Study on Behavioral and DTI Findings.” iScience 26, no. 9: 107453. 10.1016/j.isci.2023.107453. 37744405 PMC10517365

[brb370551-bib-0109] Zhang, X. , M. Yuan , S. Yang , et al. 2021. “Enriched Environment Improves Post‐Stroke Cognitive Impairment and Inhibits Neuroinflammation and Oxidative Stress by Activating Nrf2‐ARE Pathway.” International Journal of Neuroscience 131, no. 7: 641–649. 10.1080/00207454.2020.1797722. 32677581

[brb370551-bib-0110] Zhang, Y. , P. Zhang , X. Shen , et al. 2013. “Early Exercise Protects the Blood‐Brain Barrier From Ischemic Brain Injury via the Regulation of MMP‐9 and Occludin in Rats.” International Journal of Molecular Sciences 14, no. 6: 11096–11112. 10.3390/ijms140611096. 23708107 PMC3709721

[brb370551-bib-0111] Zhao, B. S. , L. X. Meng , Y. Y. Ding , and Y. Y. Cao . 2014. “Hyperbaric Oxygen Treatment Produces an Antinociceptive Response Phase and Inhibits Astrocyte Activation and Inflammatory Response in a Rat Model of Neuropathic Pain.” Journal of Molecular Neuroscience 53, no. 2: 251–261. 10.1007/s12031-013-0213-3. 24390961

[brb370551-bib-0112] Zhou, H. , Z. Zhang , H. Wei , et al. 2013. “Activation of STAT3 Is Involved in Neuroprotection by Electroacupuncture Pretreatment via Cannabinoid CB1 Receptors in Rats.” Brain Research 1529: 154–164. 10.1016/j.brainres.2013.07.006. 23880371

[brb370551-bib-0113] Zong, X. , Y. Dong , Y. Li , et al. 2020. “Beneficial Effects of Theta‐Burst Transcranial Magnetic Stimulation on Stroke Injury via Improving Neuronal Microenvironment and Mitochondrial Integrity.” Translational Stroke Research 11, no. 3: 450–467. 10.1007/s12975-019-00731-w. 31515743

[brb370551-bib-0114] Zong, X. , Y. Li , C. Liu , et al. 2020. “Theta‐Burst Transcranial Magnetic Stimulation Promotes Stroke Recovery by Vascular Protection and Neovascularization.” Theranostics 10, no. 26: 12090–12110. 10.7150/thno.51573. 33204331 PMC7667689

